# The Effect of Autologous Platelet-Rich Plasma Treatment on In Vitro Fertilization/Intracytoplasmic Sperm Injection and Its Impact on the Endometrium and Clinical Pregnancy Rate

**DOI:** 10.7759/cureus.27913

**Published:** 2022-08-12

**Authors:** Hassan S Abduljabbar, Howida Hashim, Hanin H Abduljabar, Amal A Elnaeim, Najwan H Abduljabar

**Affiliations:** 1 Obstetrics and Gynecology, Dr. Erfan and Bagedo General Hospital (EBGH), Jeddah, SAU; 2 In Vitro Fertilization, Dr. Erfan and Bagedo General Hospital (EBGH), Jeddah, SAU; 3 Obstetrics and Gynecology, King Faisal Specialist Hospital, Jeddah, SAU

**Keywords:** icsi, ivf, prp, endometrial thickness, clinical pregnancy rate

## Abstract

Background

Endometrial thickness has been identified as a prognostic factor for improving the pregnancy rate for patients with female infertility.

Study question

Does platelet-rich plasma (PRP) treatment affect the endometrial thickness and pregnancy rate after an in vitro fertilization/intracytoplasmic sperm injection (IVF/ICSI) cycle?

Aim

This study aims to evaluate the effects of autologous PRP treatment on IVF/ICSI, endometrium, and clinical pregnancy rate.

Materials, setting, and methods

This is a prospective, non-blind, randomized controlled study. The ethical committee of the Jeddah IVF Center approved the study, and informed written consent was obtained from all patients. We recruited patients who consulted at the Jeddah IVF Center from September 2020 to May 2021.

Results

A total of 70 patients undergoing IVF/ICSI and embryo transfer (ET) were randomly divided by simple randomization into two groups: those who received PRP treatment after oocyte pickup (OPU) (group A) and those who did not receive PRP treatment (control, group B). The endometrial thickness was measured after OPU and before ET. The mean ages of patients in groups A and B were 35.91 ± 4.09 (range: 24-43) and 34.63 ± 4.26 (range: 25-43), respectively, which were not statistically significant (P < 0.223). In the PRP cases, the types of infertility were primary in 16 (45.7%) and secondary in 19 (54.3%), and the causes of infertility were male factors in 24 (68.6%), unexplained in five (14.3%), ovulatory factor in two (5.6%), endometriosis in two (5.6%), tubal factor in one (2.9%), and preimplantation genetic diagnosis (PGD) in one (2.9%). In the control group, the types of infertility were primary in 14 (40%) and secondary in 21 (60%), and the causes of infertility were male factors in 21 (60%), unexplained in three (8.6%), ovulatory factor in eight (22.9%), endometriosis in one (2.8), and PGD in two (5.6%). In our study, we found that the mean endometrial thicknesses after OPU were 0.594 ± 0.089 (range: 0.4-0.7) and 0.589 ± 0.090 (range: 0.6-0.9) in the treatment and control groups, respectively (odds ratio (OR): 0.005; 95% confidence interval (CI): 0.376-0.047; P < 0.791). Before ET, the mean endometrial thicknesses were 0.86 ± 0.090 (range: 0.7-0.9) and 0.7464 ± 0.06 (range: 0.7-1) in groups A and B, respectively, (OR: 0.114; 95% CI: 0.763-0.151; P < 0.001). Of the 35 patients in each group, 12 (34.3%) and five (14.3%) had confirmed pregnancies in groups A and B, respectively (OR: 0.319; 95% CI: 0.099-1.036; P < 0.05), which is statistically significant.

Conclusion

Autologous PRP treatment in IVF/ICSI improves the endometrial thickness and clinical pregnancy rate.

## Introduction

Infertility is defined as a failure of clinical pregnancy after one year of regular unprotected sexual intercourse. Approximately 8%-15% of reproductive-aged couples worldwide suffer from infertility [1]. Infertility causes physical, emotional, and social problems in families [2]. The central issue of in vitro fertilization (IVF) treatment is a failure of implantation due to the quality of the embryo or endometrial receptivity. A thin endometrium (defined as an endometrial thickness of <7 mm) on the day of ovulation is most likely to be the cause of failure [3].

One of the treatments for recurrent implantation failure is platelet-rich plasma (PRP), a concentrated blood product rich in platelets [4,5]. In general, whole blood comprises four main components: plasma, platelets, red blood cells, and white blood cells. Plasma consists of water, protein, and dissolved salts; more than half of the blood volume is plasma, which provides the medium for all other elements transported throughout the body [6].

PRP contains multiple growth factors that typically help the body heal after an injury. Aside from accelerating wound healing, PRP also possessed anti-aging properties, which is why it has been used for the past 20 years in different areas of medicine [7]. Platelets (thrombocytes) are not only involved in the clotting process but also release other substances and growth factors. Red blood cells (erythrocytes) are involved in oxygen-carbon dioxide exchange, carrying oxygen to tissues and removing carbon dioxide from the body. White blood cells (leukocytes) are primarily responsible for immunity [8].

Although persons undergoing PRP treatment can receive PRP from a different person, this is rarely performed as PRP is usually extracted from the same individual that it will be given to. PRP extraction consists of three steps. Step 1 is withdrawing blood from the individual. Step 2 is centrifuging the obtained blood for 15 minutes. Step 3 is collecting the plasma (now rich in platelets and devoid of cellular components) in preparation for its injection [9].

The objective of this study was to identify the effect of autologous PRP treatment on IVF/ICSI and on the endometrial thickness and clinical pregnancy rate.

## Materials and methods

This is a prospective, non-blind, randomized controlled study. The ethical committee of the Jeddah IVF Center approved the study (EBGH-002 series of 2020), and informed written consent was obtained from all patients. We recruited patients who consulted at the Jeddah IVF Center from September 1, 2020, to May 1, 2021.

Sample size

The sample size was calculated based on the number of patients per six months (200) and the expected 5% repeated failure rate. The final sample size was 70 patients, which was divided into two groups.

Subjects undergoing IVF/ICSI-frozen embryo transfer (FET) were included in the study if they fulfilled the following inclusion criteria: repeated failures, age between 18 and 44 years, type of infertility eligible for IVF/ICSI, and endometrial thicknesses between 0.4 and 0.7 cm. Patients younger than 18 and older than 44 years, not eligible for IVF/ICS, who have poor embryo quality, who are not suitable for ET, who have an endometrial thickness of <0.7 cm, or with a concurrent active infection were excluded from the study.

The subjects were randomized into groups A (for PRP) and B (no PRP) via simple random sampling. The subjects’ names were drawn randomly from a pool in which each had an equal probability of being chosen. An experienced gynecologist specializing in infertility performed a transvaginal ultrasound on each subject to measure the endometrial thickness using a single machine.

PRP was prepared via a two-step centrifugation method from autologous blood.

On the day of oocyte pickup (OPU), 10-20 mL of peripheral venous blood from each subject was drawn into a syringe that contained 2.5 mL of acid citrate anticoagulant solution (Heraeus Labofuge 400 functional line + swinging rotor + four buckets with caps (75008370)) and centrifuged at 1,500 rpm for 10 minutes. The obtained plasma was then centrifuged at 3,000 rpm for five minutes to extract the PRP. Then, 0.5 mL of PRP was infused into the uterine cavity of each subject using an intrauterine insemination (IUI) catheter after the OPU.

In each group, we analyzed the following variables: age in years, type of infertility (primary or secondary), causative factor in males, unexplained, ovulatory factor, endometriosis, and tubal factor. Finally, after preimplantation genetic diagnosis, the two groups were compared.

We identified the number of embryos and the day of transfer (day 3 or day 5), and depending on embryo quality, endometrial thickness after OPU and just before the ET were measured. Then, clinical pregnancy is considered positive when patients had a positive beta-hCG test result.

The primary outcome was the endometrial thickness, and the secondary outcome was clinical pregnancy as determined by a positive serum beta-hCG test result.

Statistical analysis

The paired sample t-test and chi-square test were used for statistical analysis, and cross-tabulation was performed using the SPSS version 22 software (IBM Corp., Armonk, NY, USA). P < 0.05 was considered statistically significant.

## Results

A total of 70 patients undergoing IVF/ICSI and ET were randomly divided by simple randomization into group A, in which the subjects received PRP after OPU, and group B, in which the subjects did not receive PRP.

The mean ages of the subjects in groups A and B were 35.91 ± 4.49 years (range: 24-43 years) and 34.63 ± 4.26 years (range: 25-43 years), respectively, with the difference not reaching statistical significance (P < 0.2243). The types and causes of infertility are shown in Table 1.

**Table 1:** Patient characteristics PRP: platelet-rich plasma; PGD: preimplantation genetic diagnosis

Variable	PRP cases (n = 35)	Control (n = 35)	Odds ratio (lower-upper)	P-value
Mean age (years) (range)	35.91 ± 4.49 (24-43)	34.63 ± 4.26 (25-43)	1.05 (-0.82-3.37)	0.223
Infertility			1.263 (0.49-3.26)	0.405
Primary (%)	16 (45.7)	14 (40)		
Secondary (%)	19 (54.3)	21 (60)		
Reason for infertility				0.383
Male factor (%)	24 (68.6)	21 (60)		
Unexplained (%)	5 (14.3)	3 (8.6)		
Ovulatory factor	2 (5.6)	8 (22.9)		
Endometriosis (%)	2 (5.6)	1 (2.8)		
Tubal factor (%)	1 (2.9)	0 (0)		
PGD (%)	1 (2.9)	2 (5.6)		
Number of embryos				0.861
1 (%)	10 (28.6)	8 (22.9)		
2 (%)	11 (31.4)	12 (34.3)		
3 (%)	14 (40)	15 (42.9)		
Day of transfer				0.5
Day 3 (%)	5 (14.3)	4 (11.4)		
Day 5 (%)	30 (85.7)	31 (88.6)	1.29 (0.32-5.28)	

The mean endometrial thicknesses in groups A and B after OPU were 0.59 ± 0.089 (range: 0.4-0.7) and 0.58 ± 0.090 (range: 0.4-0.7), respectively (odds ratio (OR): 0.005; 95% confidence interval (CI): 0.0376-0.047; P < 0.791). It is not statistically significant. Before ET, the mean endometrial thicknesses in groups A and B were 0.86 ± 0.0.90 (range: 0.7-0.9) and 075. ± 0.07 (range: 0.7-1.0), respectively (OR: 0.114; 95% Cl: 0.0763-0.151; P < 0.001). In the 35 patients in each group, 12 and five had confirmed pregnancies in groups A and B, respectively (Table 2).

**Table 2:** Endometrial thickness and pregnancy rate in the PRP and control groups Number (%) OPU: oocyte pickup; ET: embryo transfer

	PRP cases (n = 35)	Control (n = 35)	Odds ratio (lower-upper)	P-value
Endometrial thickness after OPU (mean (range))	0.59 ± 0.089 (0.4-0.7)	0.58 ± 0.09 (0.4-0.7)	0.005 (0.0376-0.047)	0.791
Endometrial thickness before ET (mean (range))	0.86 ± 0.09 (0.7-0.9)	0.75 ± 0.07 (0.7-1.0)	0.114 (0.0763-0.151)	0.0001
Pregnancy (yes/no) (%)	12 (34.3%)/23 (65.7%)	5 (14.3%)/30 (85.7%)	0.319 (0.099-1.036)	0.05

## Discussion

Intrauterine PRP infusion is used to treat the endometrium. PRP is rich in platelets; it is derived from blood plasma, which is prepared from fresh whole blood. PRP therapy may potentially improve a thin endometrium that is unresponsive to conventional treatment and represents a new method for the thin endometrium with poor response.

It has been reported that PRP promotes endometrial growth and improves pregnancy outcomes in patients with a thin endometrium [10]. Do we know the suitable endometrial thickness for conceiving? The endometrial thickness is positively correlated with the number of pregnancy losses and live births in IVF. Hence, to increase the chances of live birth and minimize pregnancy loss, the optimal endometrial thickness should be >7 mm [11].

The adverse effects of PRP therapy include pain at the injection site, infection, allergic reaction, hematoma, and skin discoloration [12].

Our study aimed to analyze the effect of intrauterine PRP therapy in subjects undergoing IVF/ICSI or FET cycles. The effect of PRP treatment on endometrial thickness and clinical pregnancy was determined and recorded. In our study, we found that the mean endometrial thicknesses after OPU were 0.594 ± 0.089 and 0.589 ± 0.090 in the treatment and control groups, respectively; the difference between groups was not statistically significant (P < 0.791). Meanwhile, the mean endometrial thicknesses before ET were 0.86 ± 0.094 and 0.746 ± 0.066 in the treatment and control groups, respectively; the difference was statistically significant (P < 0.0001). The effectiveness of intrauterinePRP treatment in improving a thin endometrium was studied in the trial, and the results show that PRP treatment results in thicker endometrium in patients with a thin endometrium that is refractory to treatment [13]. PRP treatment also enhances the growth of endometrium and pregnancy outcomes in women with a thin endometrium [14]. Evaluation of the effectiveness of PRP in treating a thin endometrium was tried, and it was found to improve endometria. In addition, PRP treatment also improved implantation, pregnancy, and live birth rates of subjects with a thin endometrium [15].

In infertile women with a history of failed implantation, intrauterine PRP infusion before frozen ET was found to have no significant effect on pregnancy [16]. A prospective cohort with a large number of patients is necessary to explore the underlying mechanisms responsible for PRP’s beneficial effects and further investigate the benefits of PRP treatment in women with a thin endometrium who have undergone frozen ET (FET). Chang et al. found that PRP improves endometrial proliferation, embryo implantation rate, and clinical pregnancy rate in women with a thin endometrium after FET [10].

## Conclusions

Autologous PRP treatment in IVF/ICSI improves the endometrium and clinical pregnancy rate. Although there is increasing evidence suggesting the possible benefit of PRP treatment on the endometrium of infertile women, the mechanism is not very clear and needs further explication. Further, careful studies, especially RCTs, on larger scales are required, and follow-ups on long-term health and complications are needed.
